# The Impact of Load Style Variation on Gait Recognition Based on sEMG Images Using a Convolutional Neural Network

**DOI:** 10.3390/s21248365

**Published:** 2021-12-15

**Authors:** Xianfu Zhang, Yuping Hu, Ruimin Luo, Chao Li, Zhichuan Tang

**Affiliations:** 1 Schlool of Jewelry and Art Design, Wuzhou University, Wuzhou 543002, China; zhangxianfu@zju.edu.cn (X.Z.); 3779@hnsyu.edu.cn (Y.H.); 2 College of Computer Science and Technology, Zhejiang University, Hangzhou 310027, China; joeluo@zju.edu.cn (R.L.); superli@zju.edu.cn (C.L.); 3 Industrial Design Institute, Zhejiang University of Technology, Hangzhou 310023, China

**Keywords:** sEMG image, load style, gait recognition

## Abstract

Surface electromyogram (sEMG) signals are widely employed as a neural control source for lower-limb exoskeletons, in which gait recognition based on sEMG is particularly important. Many scholars have taken measures to improve the accuracy of gait recognition, but several real-time limitations affect its applicability, of which variation in the load styles is obvious. The purposes of this study are to (1) investigate the impact of different load styles on gait recognition; (2) study whether good gait recognition performance can be obtained when a convolutional neural network (CNN) is used to deal with the sEMG image from sparse multichannel sEMG (SMC-sEMG); and (3) explore whether the control system of the lower-limb exoskeleton trained by sEMG from part of the load styles still works efficiently in a real-time environment where multiload styles are required. In addition, we discuss an effective method to improve gait recognition at the levels of the load styles. In our experiment, fifteen able-bodied male graduate students with load (20% of body weight) and using three load styles (SBP = backpack, SCS = cross shoulder, SSS = straight shoulder) were asked to walk uniformly on a treadmill. Each subject performed 50 continuous gait cycles under three speeds (V3 = 3 km/h, V5 = 5 km/h, and V7 = 7 km/h). A CNN was employed to deal with sEMG images from sEMG signals for gait recognition, and back propagation neural networks (BPNNs) and support vector machines (SVMs) were used for comparison by dealing with the same sEMG signal. The results indicated that (1) different load styles had remarkable impact on the gait recognition at three speeds under three load styles (*p* < 0.001); (2) the performance of gait recognition from the CNN was better than that from the SVM and BPNN at each speed (84.83%, 81.63%, and 83.76% at V3; 93.40%, 88.48%, and 92.36% at V5; and 90.1%, 86.32%, and 85.42% at V7, respectively); and (3) when all the data from three load styles were pooled as testing sets at each speed, more load styles were included in the training set, better performance was obtained, and the statistical analysis suggested that the kinds of load styles included in training set had a significant effect on gait recognition (*p* = 0.002), from which it can be concluded that the control system of a lower-limb exoskeleton trained by sEMG using only some load styles is not sufficient in a real-time environment.

## 1. Introduction

The surface electromyogram (sEMG) signal is employed widely as a neural control source for lower-limb exoskeleton and prosthesis devices [1,2]; these sEMGs are generated by the electrical activity of the muscle fibre during contraction or relaxation and can be obtained from the skin surface in a noninvasive manner by a muscle-computer interface (MCI) communication system [3]. Assisted by the MCI, the reflections of the sEMG at the level of muscle activity are transformed into interactive commands that express the user’s movement intentions for the control of lower-limb exoskeletons [2,4]. The control signals that are used to guide the work of lower-limb exoskeletons can be derived from gait recognition from sEMG signals [5]. Joshi, C. D et al. [1] used the sEMG signal from the lower-limb muscles based on a Bayesian information criteria algorithm to recognize eight different gait phases for the control of exoskeletons. Chen Lingling et al. [6] employed sEMG from leg muscles to recognize the gait phases and translated it as a switch signal of self-locking control to drive the lower-limb prostheses. Xu et al. [7] used the sEMG signals from the calf muscle as the input of gait recognition (including go forward, go backward, turn left, and turn right), and then the gait information was leveraged to control the motion of the lower-limb exoskeleton and realize human–computer interaction. Peng et al. [2] proposed an SVM-based gait recognition method using sEMG signals to control a lower-limb prosthesis device.

The above literature focuses mainly on increasing the gait recognition performance based on offline sEMG signals by multisensor cooperation [8], algorithm optimization [9], the incorporation of more reasonable features [10], etc., to control lower-limb exoskeletons and prostheses more effectively. Some studies have obtained an ideal gait recognition accuracy of up to 95% in laboratory conditions [11], but there are still several real-time limitations in using sEMG signals to recognize gait for the control of lower-limb exoskeletons and prostheses. For example, compared with the laboratory, there are more non-constant variations that may greatly affect the gait recognition performance based on sEMG in a real-time environment, such as the instability of sEMG signal [12], inaccurate positioning of the electrodes placed on the muscles [13], slope variations on the ground [14], and the different loads that user can carry [15]. In addition, the different load styles are also critical variables which can often be encountered in real-time environments. In the laboratory, the training data are obtained under a determined load style, and the employed training strategy of gait recognition has the ability to identify the style of the load. However, in real-time conditions, the load styles we choose were random, which is significantly different from the conditions in the laboratory. Many scholars have conducted in-depth studies of the effects of different load styles on mechanical parameters and gait characteristics. For example, Abaraogu, U. O. et al. [16] exposed the effects of a 1-strap backpack load and a 2-strap backpack load on the gait phases and perceived the exertion of young adults. Dahl, K. D. et al. [17] compared the traditional backpack and the nontraditional BackTpack and revealed that the subjects who use the nontraditional BackTpack can obtain a more natural stance and gait pattern. Pascoe, David D. et al. [18] investigated the effects of different styles of carrying book bags on the gait kinematics of youths and found that book bags carried on one shoulder can significantly change gait and posture compared with a two-strap backpack under daily physical stresses. However, few scholars have paid attention to the effects of different load styles on the accuracy of gait recognition based on sEMG, so this area is still not well understood.

Algorithms, such as BPNN [19], Long Short-Term Memory (LSTM), SVM [2], and linear discriminant analysis (LDA) [1], are combined with manual feature extraction for gait recognition. Manual feature extraction often loses some features [4], resulting in class performance that is not very satisfactory.

Recently, CNN have achieved great success in computer vision [20], image processing [21], speech recognition [22], and other fields. Subsequently, CNN have been applied successfully to process sEMG signals [4,23]. For example, Park and Lee [4] employed a CNN model to learn the features of six hand movements based on sEMG, and the performance of the CNN was better than that of the SVM. Fricke C. et al. [23] applied CNN to process sEMG for gait recognition and achieve a better result than classic classification algorithm. With the deepening of research, some scholars have further improved CNN for recognition and classification with promising results. Chen, Cf. et al. [24] proposed a method based on LSTM-CNN for gait recognition and obtained 97.78% accuracy. Guo B. et al. [25] designed a light-weight convolutional neural network (Lw-CNN) for sEMG to recognize the upper-limb motion intents, and the average accuracy is up to 95%.

Inspired by the success of EMG maps [26,27] and myoelectric topography [28,29] in applications of sEMG-based pattern recognition, as well as the advantage of CNN in image processing, some scholars have tried to convert high-density sEMG (HD-sEMG) signals into greyscale images. Under these conditions, EMG-based pattern recognition can be accordingly transformed to image classification. Geng, W. et al. [30] trained a deep CNN model using sEMG images converted from HD-sEMG signals to recognize eight gestures and obtained 99.0% accuracy under 40 frames of sEMG images at a 1000 Hz sampling rate. With the same thinking, Yu Du [31] presented an eight-layer convolutional neural network based on adaptation architecture to deal with HD-sEMG images for gesture recognition, and the results outperformed four other conventional classifiers in both intra- and inter-session tests. The applications of sEMG images in gesture recognition in the existing literature are mainly focused on HD-sEMG. For sEMG images from sparse multichannel sEMG (SMC-sEMG) signals, which require precise anatomical positioning, there is no relevant research in the field of gait recognition. sEMG images do not lose their inherent features, which are commonly lost in manual feature extraction, and the SMC-sEMG can also be converted to an sEMG image as an HD-sEMG. Moreover, implementing the CNN on a small-size dataset can achieve good performance if the choice of filters, weights and number of layers are reasonable [32]. Thus, it is also reasonable to study gait recognition based on SMC-sEMG images and CNNs.

To clarify these issues thoroughly, fifteen adult males took part in our experiment. Every subject walked on a uniform running treadmill with three load styles under three speeds. The CNN was employed to deal with the SMC-sEMG image from the SMC-sEMG signal to investigate (1) whether the different load styles have a significant impact on gait recognition; (2) whether the CNN based on SMC-sEMG images performs well in gait recognition; and (3) whether a lower-limb exoskeleton (or prosthesis) control system trained by sEMG signals from single or parts of load styles still functions well in the face of multiload style conditions.

## 2. Experimental Method

### 2.1. Subjects

Fifteen able-bodied (intact) male graduate students (mean ± SD; age = 26 ± 2 years, height = 172.2 ± 5.4 cm, weight = 64.1 ± 4.2 kg, Body Mass Index (BMI) = 21.6 ± 0.7 kg/m^2^) from Zhejiang University who were skilled in using a treadmill participated in our study. Before the experiment, all subjects underwent anthropometric measurements, including age, height, and weight. Then, they were given a complete physical examination to ensure that they were free from neurological and musculoskeletal diseases. They were not allowed to participate in any physical strenuous exercise that might introduce fatigue. The purpose, details, and procedure of the experiment were provided to the local human ethical clearance committee of Zhejiang University for review and were approved. All subjects provided informed consent after they completely understood the experiment.

### 2.2. Experimental Procedure

**Figure 1:**
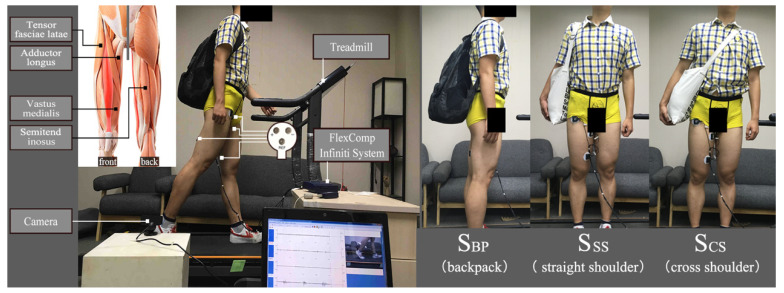
The experimental equipment and connection, the placement of the electrodes, and load styles (S_BP_ = backpack, S_CS_ = cross shoulder, and S_SS_ = straight shoulder).

According to the different roles the lower-limb muscles play during the gait cycle, four thigh muscles (tensor fasciae latae, semitendinosus, adductor longus, and vastus medialis) were selected for the acquisition of the surface EMG signal; of these, the tensor fasciae latae and the semitendinosus are closely related to the stance phase, while the adductor longus and the vastus medialis mainly affect the swing phase [33,34], as shown in Figure 1.

In the experiment, all the subjects were asked to wear T-shirts and white shoes to strengthen the colour contrast between their shoes and the treadmill belt, which is good for the division of the gait phase by identifying the demarcation points of the heel strike and toe off of both feet. Then, the subjects were familiarized with the entire experimental process and the equipment.

Four electrode groups were used to acquire sEMG signals, every group contained 3 Ag/AgCl electrodes. Each electrode group was adhered to a target muscle, and the line through the two electrodes (non-reference electrode) aligned with the direction of target muscle fibres on the midline of the muscle belly [35]. Then, the exact location on which the electrode groups were placed on the skin was marked to ensure that the electrode groups were placed in the same position in each experiment, and a new electrode group was used in each experiment. To improve the stability of the signal and the conductivity of electrodes, conductive gel was used after the hair on the four measured muscles was shaved off, and the skin was cleaned with medical alcohol [36]. We also used medical bandages to fix data wires on the thigh to reduce the noise signal produced by shaking wires, and the bandage should not be loose or too tight, so that it could fix the wire without interfere with muscle activity. In addition, the subject rested for 10 min after each experiment to remove the negative effects from fatigue.

After all instruments were configured correctly and the signal was checked to be in a very good steady state, the experiment continued to the next step. The subjects were required to walk on the treadmill at three uniform speeds (V3, V5, and V7 = 3, 5, and 7 km/h, respectively) [37] under three load styles (S_BP_, S_CS_, and S_SS_; Figure 1) for 50 continuous gait cycles (as shown in Figure 1). The weight of the load (bag and load) was 20% of the body weight of the subject [36]. In the entire experiment, 6750 data sets (15 subjects × 50 gait cycles × 3 load styles × 3 speeds) were acquired, as shown in Table 1.

In the experiment, we chose a traditional one-shoulder bag and backpack with no sternum strap or hip-loading belt to carry the load. The adoption of this bag and backpack makes the experiment more convincing for their universal applicability. Then, the shoulder straps of the backpack were adjusted to ensure that the load was placed on the level of pelvis [17], and the same method was employed to ensure that the load of the shoulder bags was placed at the same level height.

### 2.3. Data Acquisition

The subjects were instructed that data would be collected when they walked steadily on the treadmill. The surface electromyographic (sEMG) signals of the target muscles of subjects were collected by MyoScan-Pro sensors, which have the ability to record up to 1600-microvolt (μV) sEMG signals. The collected signal was filtered in a range of 10 Hz to 400 Hz, and a 50 Hz notch filter was employed to remove line interference. Then, the signals were input into a PC for data analysis using a digital sEMG system (FlexComp Infiniti System, Thought Technology Ltd., Montreal, QC, Canada), which contains ten channels and a video collection system [36], which can guarantee the synchronism of the sEMG data and the video data. In the experiment, all sEMG signals were sampled at 2000 Hz.

The video data were collected synchronously by a high-speed, high-resolution camera at 60 frames per second. The camera was fixed perpendicular to the direction of the running belt of the treadmill, and the vertical distance to the treadmill was adjusted to ensure that the entire running belt was in the camera’s frame [18].

### 2.4. Signal Preprocessing

All sEMG and video signals were processed offline. The synchronized video of each experiment was segmented into independent gait cycles one by one, and then each gait cycle was subdivided into 5 separate gait subphases (initial stance, midstance, terminal stance, initial swing, and terminal swing) by a trained and experienced observer by identifying the demarcation point of the heel strike and toe off of both feet [38]. Then, the divided video was used as a reference to label the five gait subphases of the synchronized sEMG signal [5]. The labelled sEMG data were then segmented to extract features by the overlapped windowing technique [39], and the analysis window was set to 30 ms and overlapped by 10 ms.

### 2.5. Classification Methods

#### 2.5.1. CNN

1. sEMG image.

The sample size of the sEMG signal after pre-treatment was 240 (4 channels for sEMG signal acquisition, analysis window of 30 ms, sample rate of 2000 Hz). The sEMG signal of each analysis window was recombined and arranged into a 4 × 60 greyscale image to take advantage of the CNN in image processing for feature extraction and classification.

2. CNN structure.

**Figure 2:**
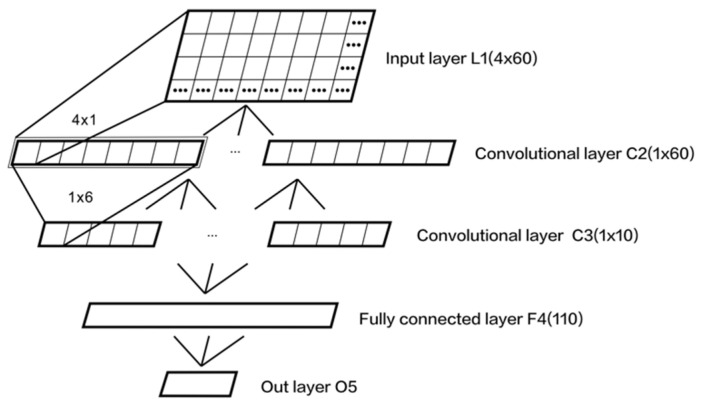
CNN topology.

We structured the CNN as shown in Figure 2, which included one input layer (L1), 2 convolution layers (C2 and C3), and 2 fully connected layers (F4 and O5).

In this paper, a one-dimensional convolution operation was used, and the neurons were defined as (*l*, *m*, *r*, and *f*), in which *l* was the layer, *m* was the feature image, *r* was the specific position of neurons in the feature image, and *f* was the activation function. The input and out of a neuron were recorded as $x_{m}^{l}\left(r\right)$ and $P_{m}^{l}\left(r\right),\;\mathrm{respectively}$, and $P_{m}^{l}\left(r\right)=f\left(x_{m}^{l}\left(r\right)\right)$. In C2 and C3, we employed *f* (*u*) *=* α *tanh* (β *u*) as the activation function (where α = 1.7159 and β = 2/3 [40]). In F4 and O5, we used the sigmoid function $f\left(u\right)=1/(1+exp^{-u}$) as the activation function. Each layer is expressed in detail as follows:

(1) L1: input layer. the input matrix *H_C,T_* (*C* = 4, channels; *T* = 60, sample points), which can be expressed as 4 × 60.

(2) C2: convolutional layer. The aim of this layer was to carry out spatial filtering for input signals. We used 7 spatial filters and set the convolution kernel [4 × 1] in the layer, so 7 feature maps (the size of each feature map was [1 × 60]) were obtained. The function can be expressed as (1) [41].

(1) $$P_{m}^{2}=f\left(\overset{i\leq 4}{\underset{i=1}{\sum }}H_{i,r}\times K_{m}^{2}+b_{m}^{2}\left(r\right)\right)$$

where $K_{m}^{2}$ is the convolution kernel of C2, and $b_{m}^{2}\left(r\right)$ is the bias of C2.

(3) C3: which was similar to C2, which filtered input signals in the time domain. We used 6 time filters and set the convolution kernel size [1 × 6], and 42 feature maps (each size [1 × 10]) were obtained. The function can be expressed as (2) [41].

(2) $$P_{m}^{3}=f\left(\overset{i\leq 6}{\underset{i=1}{\sum }}P_{m}^{2}\left[\left(r-1\right)\times 6+i\right]\times K_{m}^{3}+b_{m}^{3}\left(r\right)\right)$$

where $\;K_{m}^{3}$ is the convolution kernel of C3 and $b_{m}^{3}\left(r\right)$ is the bias of C3.

(4) F4: fully connected layer, which is fully connected with C3, and 110 neurons were set in the layer.

(3) $$P^{4}=f\left(\overset{i\leq 42}{\underset{i=1}{\sum }}\overset{z\leq 10}{\underset{z=1}{\sum }}P_{i}^{3}\left(z\right)w_{i}^{4}\left(z\right)+b^{4}\left(r\right)\right)$$

where $w_{i}^{4}\left(z\right)$ is the weight from C3 to F4 and $b^{4}\left(r\right)$ is the bias.

(5) O5: outer layer, which was fully connected to F4 and included 5 neurons (5 gait phases: initial stance, midstance, terminal stance, initial swing, and terminal swing). The expression is

(4) $$P^{5}=f\left(\overset{i\leq 110}{\underset{i=1}{\sum }}P^{4}\left(i\right)w^{5}\left(i\right)+b^{5}\left(r\right)\right)$$

where $w^{5}\left(i\right)$ is the weight from F4 to O5, and $b^{5}\left(r\right)$ is the bias.

In our CNN, gradient descent was employed to adjust the weight and bias to ensure the smallest error. The model’s loss convergence was determined to be optimal after 10^4^ iterations. The range of input weight and bias was $[-1/n{\left(l.m.r\right)}_{in}$, $1/n{\left(l.m.r\right)}_{in}$], in which $n{\left(l.m.r\right)}_{in}$ was the number of neurons in the upper layer connected with the *i*th neuron in this layer. The learning rates $\sigma_{2–3}$ and $\sigma_{4–5}$ (C2–C3 and F4–O5, respectively) were defined by (5) and (6) [41,42].

(5) $$\sigma_{2–3}=\frac{2\psi }{N_{n\left(l.m.0\right)}^{sh}\sqrt{N_{n\left(l.m.i\right)}^{in}}}$$

(6) $$\sigma_{4–5}=\frac{\psi }{\sqrt{N_{n\left(l.m.i\right)}^{in}}}$$

where $N_{n\left(l.m.0\right)}^{sh}$ is the number of neurons sharing weight, $N_{n\left(l.m.i\right)}^{in}$ is the number of inputs, and ψ is a constant.

#### 2.5.2. Classic Classification Algorithm

In contrast to a CNN, an SVM and BPNN were employed to recognize the gait phases.

*A: Feature Extraction*

After filtering, labelling, and segmentation, the sEMG data were extracted to reduce the sEMG signal dimension, which decreased the complexity and improved the computational efficiency of pattern recognition and classification. Based on the same thinking, it was important to choose features with clear distinction, less complexity, and substantial efficiency [43]. In our paper, integral electromyography (*iEMG*) and the root mean square (*RMS*) were employed as the input features of SVM and BPNN, and they can be expressed as shown in Equations (7) and (8).

(7) $$iEMG=\frac{1}{N}\overset{N}{\underset{k=1}{\sum }}\left|V_{k}\right|$$

(8) $$RMS=\sqrt{\frac{1}{N}\overset{N}{\underset{k=1}{\sum }}V_{k}^{2}}$$

where $V_{k}$ is the *i*th sEMG data sampling. *k* = 1, 2…, *N*. *N* is the number of sampling points in each time window.

Therefore, the input vector *P* can be expressed as

*P_k_* = {*RMSk1*, *iEMGk1*, *RMSk2*, *iEMGk2*, *RMSkr*, *iEMGkr*}, where *k* is the number of time windows and *r* is the number of sEMG channels.

In this experiment, the sEMG data were collected from four muscles, which means that each time window included eight features. The outputs were five gait phases, i.e., initial stance, midstance, terminal stance, initial swing, and terminal swing.

*B: Classification Methods*

The SVM is widely used to process EMG signals. With appropriate kernel functions, the SVM can obtain good adaptability and robustness. Therefore, in this study, we chose the Gaussian radial basis function (RBF) as the kernel function to construct the SVM model [44]. Cross-validation was employed in the training phase of SVM model construction.

BPNNs have achieved great success in pattern recognition and classification based on sEMG signals. In our study, we designed a three-layer BPNN, which includes 20 nodes in the hidden layer. This means that there were eight nodes in the input layer and one node in the output layer, which was the gait phase corresponding to the time window.

### 2.6. Data Analysis

To study the impact of load styles on gait recognition, we converted the sEMG signal into an sEMG image from different load styles and speeds (9 experiments per subject, 135 experiments in total) and took the sEMG image as the input of the CNN model. In each experiment, 60% of the data were selected randomly for training CNN models, 20% of the data were set for selecting optimal parameters, and the remaining 20% of the data were set to test the performance. In each experiment, the recognition accuracies of the five gait phases were obtained separately, and the five values were averaged to acquire the average recognition accuracy of a single trial. For comparison, the SVM and BPNN were employed to process the same data.

Univariate analysis of variance (UNIANOVA) was adopted to investigate the impact of load styles on gait recognition. The least significant difference (LSD) post hoc test was implemented to search for the full effect if the impact was significant. SPSS 24.0 (SPSS Inc., USA) was employed to analyse the experimental data, and a confidence level of 95% was selected.

## 3. Results

### 3.1. Accuracies of the Five Gait Phases under Different Load Styles and Speeds

Three different CNN models were constructed under each speed, as shown in Table 2. There were substantial differences in the accuracy of gait recognition. The highest value (97.56%) appeared at S_BP_ under V5, and the lowest value (80.73%) was from S_CS_ under V3. When only the load style was considered, the highest accuracy of gait recognition at three different speeds came from S_BP_ (88.60% at V3, 97.56% at V5, and 93.65% at V7), followed by S_SS_ (85.17% at V3, 94.67% at V5, and 91.11% at V7), and the lowest value occurred at S_CS_ (80.73% at V3, 87.96% at V5, and 85.54% at V7). The gait recognition total average accuracies at the three load styles and three speeds were significantly different. V5 had the highest value (93.40%), and V3 had the lowest value (84.83%).

UNIANOVA revealed that the different load styles had a substantial effect on gait recognition (*p* < 0.001); the same conclusion was also drawn from the speed data. In addition, the LSD post hoc test showed a significant difference among the three load styles (*p* < 0.05).

**Figure 3:**
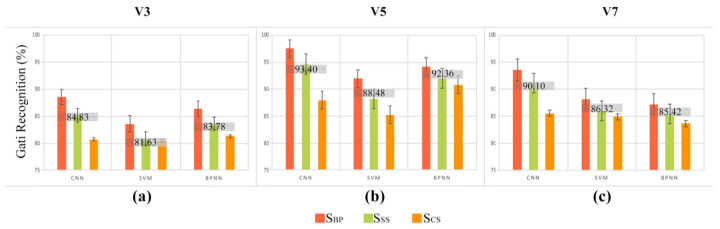
Gait recognition comparisons among three load styles with three algorithms at three speeds (V3 (**a**), V5 (**b**) and V7 (**c**)).

For comparison, we adopted an SVM and BPNN to process the same data. The gait recognition performance of the three algorithms is presented in Figure 3. Under the three speeds, the average gait recognition performance of the three load styles from the CNN was the best (84.83% at V3, 93.40% at V5, and 90.10% at V7). Furthermore, the performance of gait recognition from the SVM and BPNN indicated the same tendency: the performance from S_BP_ was better than that from S_SS,_ and the performance of S_CS_ was the worst among the three algorithms under each speed for all experiments. UNIANOVA showed the same conclusion: the different load styles also had significant effects on gait recognition, as obtained from SVM and BPNN (SVM: *p* < 0.05; BPNN: *p* < 0.05).

### 3.2. Results of the Confusion Matrix

**Figure 4:**
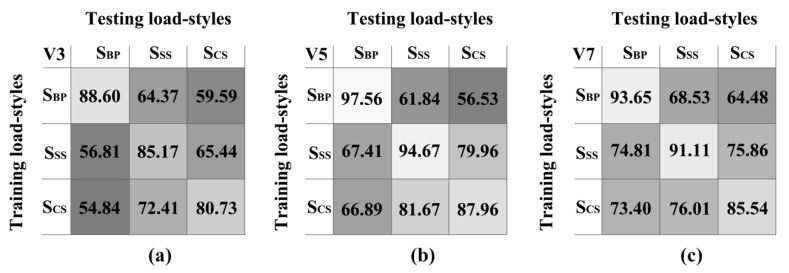
Gait recognition (%) matrices under three speeds (V3 (**a**), V5 (**b**) and V7 (**c**)). Each entry value in the matrix represents a gait recognition performance (%) from the indicated training load style (vertical axis) and testing load style (horizontal axis). A lighter colour means better performance.

Three different CNN models were constructed at each speed, which were trained by the data from one of the three load styles, and the test data were from all three load styles evaluated one by one at the same speed. All the performances are presented in Figure 4. Every value in those matrices represented gait recognition performance among all subjects according to the designated training load styles (vertical axis) and testing load styles (horizontal axis). The values on the main diagonal were obtained when the training and testing data were from the same load styles (intra-load styles), and the other off-main diagonal values were obtained when the training and testing data were from different load styles (inter-load styles). Then, a *t*-test indicated that there was a marked difference (*p* < 0.05) between the values on and off the main diagonal, which means that load styles had a significant effect on gait recognition. For example, when the testing data were from S_BP_ and the training data were from S_BP_, S_CS_, and S_SS_ under V5, the gait recognition performances (97.56%, 66.89%, and 67.41%, respectively) were markedly different. A similar phenomenon was also found in other experiments. Among all the experiments, the best and poorest performances at V3 appeared when the training data came from S_BP_ and S_CS_ and the testing data came from S_BP_ (S_BP_–S_BP_: 88.60%; S_CS_–S_BP_: 54.84%). For V5 and V7, the best and poorest performances appeared when the training data came from S_BP_ and the testing data came from S_BP_ and S_CS_ (S_BP_–S_BP_: 97.56% at V5 and 93.65% at V7; S_BP_–S_CS_: 56.53% at V5 and 64.48% at V7).

Statistics were employed to analyse all the values in the three matrices, and the intra- and inter-load styles and overall accuracy, expressed as the mean ± SD, are presented in Table 3. The total intra-load style accuracy was 89.44%, which was much better than the total inter-load style accuracy (67.83%) and the total overall accuracy (75.03%). Moreover, as shown in Table 2 and Figure 4, different speeds had a substantial effect on gait recognition. The overall gait recognition accuracy at V3 (69.77%) was significantly different from those at V5 (77.17%) and V7 (78.15%).

### 3.3. Results of the Mixed-Load Styles Evaluation

**Figure 5:**
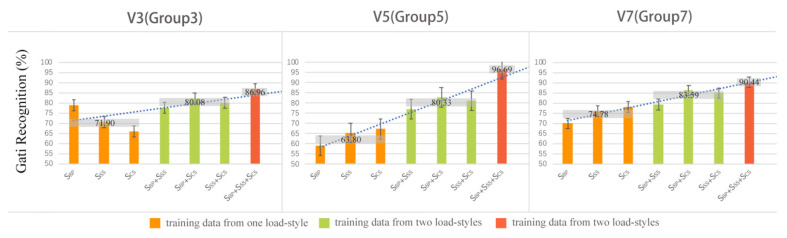
The average accuracies of the gait recognition (%) for one, two, three, and four loads at each speed are labelled in the figure.

At each speed, all the data from the three load styles were mixed as the testing data group (group 3 (V3), group 5 (V5), and group 7 (V7)), and the training data were mixed as one or several load styles. This means that there were seven different combinations of load styles in the training data at each speed: one load style in the training data (three kinds), two load styles in the training data (three kinds), and three load styles in the training data (one kind). The performances are summarized in Figure 5, which shows that the poorest performance occurred when the training set included one load style (71.90% at V3, 63.80% at V5, and 74.78% at V7), while the performance was much better when the training set included two load styles (80.08% at V3, 80.33% at V5, and 83.59% at V7); the best performance occurred when the training set included three load styles (86.96% at V3, 96.69% at V5, and 90.44% at V7). The trend lines (linear prediction) intuitively display the marked impact of the kinds of load styles included in the training data on gait recognition.

## 4. Discussion

In this study, we first investigated the impact of load styles on gait recognition based on sEMG images from sEMG signals using a CNN. Then, we discussed whether a lower-limb exoskeleton control system with sEMG as the control signal and trained by parts of the load styles can still function efficiently in real-time conditions where a multiload style is required.

Table 2 shows that the gait recognition performances among the three load styles under each speed fluctuate substantially (88.60–80.73% at V3, 97.56–87.96% at V5, and 93.65–85.54% at V7). Then, UNIANOVA suggested that there was a significant effect of the load styles on gait recognition (*p* < 0.001), and the LSD post hoc test showed that a significant difference existed among the three load styles (*p* < 0.05). The load had a marked effect on the muscle activity, and the different load styles could influence the activity levels of muscles involved, which may influence the fatigue of the corresponding muscles and the gait stability [45], which would then affect gait recognition. Simpson et al. [46] revealed that a one-strap bag (S_SS_, S_CS_) had a markedly greater energy cost than a S_BP_ [18], indicating that the fatigue under the one-strap bag was much worse than that under the backpack, which is in accordance with our findings that the gait recognition performances of S_CS_ and S_SS_ were much worse than that of S_BP_. Different load styles had significant effects on posture and gait balance [17]. With S_SS_ and S_CS_, the load was placed on one side of the body, which caused much greater lateral spinal bending and worse balance than with S_BP_ [18], and the marked force difference from the load on the left and right sides of the body also substantively damaged the gait balance [47]. Moreover, without physical hindrance, the swing amplitudes of the bags of S_SS_ and S_CS_ were much greater than that of S_BP_ at the same speed, which leads to worse physical stability [48]. Yu-Chih Hung et al. [49] revealed worse posture stability with a one-shoulder bag (i.e., S_CS_, S_SS_ in our study) than that with a S_BP_, which can explain to some extent why the gait recognition results of S_SS_ and S_CS_ were worse than that of S_BP_.

The SVM and BPNN algorithms were employed to deal with the same data separately to verify our conclusion, and the results shown in Figure 3 showed that load styles had a significant effect on gait recognition, which was also clearly obtained from SVM and BPNN (*p* < 0.05); however, the performances of SVM and BPNN were generally worse than that of the CNN at the three tested speeds. A possible reason for this difference was that no information was lost in the feature exaction of the CNN, while the hand-crafted features (*iEMG*, *RMS*) in the SVM and BPNN may drop inherent information [4], resulting in the poor performance of gait recognition. The results demonstrate that in addition to the HD-sEMG image, the CNN based on the SMC-sEMG image also performs well in gait recognition.

To explore whether lower-limb exoskeletons still work well in a real-time environment, a feasible method was to test whether the control system using sEMG as a control signal was sufficiently effective when the training and testing load styles were unequal (i.e., inter-load styles) [12]. Our experiment (Table 3) showed that when the testing and training data were from the same load styles at each speed (intra-load styles), the performance of gait recognition was much better than the performance of gait recognition when the data were from different load styles (inter-loads) (84.83–62.24% at V3, 93.40–69.05% at V5, 90.10–75.51% at V7, and total 89.44–69.94%). Therefore, we can safely conclude that a control system of lower-limb exoskeletons trained by single or partial load styles is obviously insufficient in a real-time environment where various load styles are required.

We further studied the impact of load styles on gait recognition in real-time environments and explored ways to increase gait recognition at the level of load styles. All the data at each speed were collected as a group for the testing set to imitate a real-time environment, in which varied load styles are the norm. The three datasets from the three load styles were freely combined into seven datasets as a training set to imitate the possible load styles that people encounter in real environments. The performance of gait recognition at each speed revealed the same conclusion: if more load styles are included in the training set, better performance is achieved, as shown in Figure 5. UNIANOVA suggested that the kinds of load styles included in the training set had a significant effect on gait recognition (*p <* 0.001), and based on these results, we can conclude that increasing the kinds of load styles in the training set can clearly improve gait recognition and can subsequently improve the effectiveness of the exoskeleton using the EMG signal as a control signal in a real-time environment.

In addition, we found that speed was not a factor that could be ignored in gait recognition, as shown in Table 2. The total average gait recognition at V5 was best (93.40%), followed by that at V7 (90.10%), and the lowest performance was at V3 (84.83%). These results were consistent with those of Rossi et al. [37], who found that the gait at a fast speed was better than that at a slow speed. The performance of gait recognition at V5 was better than that at V7. A possible reason is that V5 was a natural walking speed in daily life, which is more likely to produce ideal and stable sEMG signals [50]. Kadaba et al. [51] demonstrated that a normal and comfortable pace was good for the repeatability of sEMG in gait cycles and was good for improving gait recognition performance.

However, some limitations are present in our experiments. For instance, the subjects in our experiments were all able-bodied people who were not afflicted by stroke or hemiplegia (the real users of exoskeletons). Second, we only explored whether the CNN based on SMC-sEMG images performs well in gait recognition and investigated the impact of different load styles on the accuracy of gait recognition. However, we did not validate the control system based on sEMG with the exoskeleton on. Therefore, the next study will focus on the inclusion of people with disabilities beside verification of sEMG control system with the exoskeleton on. With these factors taken into consideration in studies on the effects on gait recognition, we will attempt to improve the accuracy of gait recognition and the effectiveness of the control system for lower-limb exoskeletons based on sEMG in real-time environments.

## 5. Conclusions

In this paper, we demonstrated that the different load styles had marked effects on gait recognition based on sEMG images using a CNN, and the same conclusion was obtained from a BPNN and SVM, which means that the marked effect was not mainly caused by algorithms but load styles. In our study, the gait recognition based on SMC-sEMG images using the CNN was much better than those using the SVM and BPNN, indicating that the CNN has good performance in processing SMC-sEMG images for gait cognition in addition to HD-sEMG images. We also found that the performance of gait recognition when the training and testing data of CNN were from intra-load styles was much better than the performance when they were from inter-load styles. Moreover, we freely combined the data from three load styles at each speed to imitate the possible load styles that the users of lower-limb exoskeletons may meet in real-time environments and pooled all data at each speed as a group to imitate a real-time environment at the level of the load styles. The results showed that if more load styles were included in the training data, better gait recognition performance was obtained, which suggests that a lower-limb exoskeleton control system trained by a single load style or by only some load styles does not work efficiently in real-time environments. We can also deduce that including more load styles in the training data was an effective method to improve gait recognition in a real-time environment at the level of the load styles. This study not only contributes to the EMG guidance of a lower-extremity exoskeleton but, at a certain level, also explains that the difference in load styles significantly impacts the posture and gait balance of the human body, providing good guidance for the posture correction of the human body, especially for teenagers.

## Figures and Tables

**Table 1:** Speed and load style information in nine trials.

Speed	Load-Style	Trial
**V3**	S_BP_: backpack	Trial 1
	S_SS_: straight shoulder	Trial 2
	S_CS_: cross shoulder	Trial 3
**V5**	S_BP_: backpack	Trial 4
	S_SS_: straight shoulder	Trial 5
	S_CS_: cross shoulder r	Trial 6
**V7**	S_BP_: backpack	Trial 7
	S_SS_: straight shoulder	Trial 8
	S_CS_: cross shoulder	Trial 9

**Table 2:** Gait phase recognition (%), gait recognition (%) under each load style and speed, and the total average values of gait recognition (%) at three load styles and three speeds.

Speed	V3			V5			V7		
**Load styles**	**S_BP_**	**S_SS_**	**S_CS_**	**S_BP_**	**S_SS_**	**S_CS_**	**S_BP_**	**S_SS_**	**S_CS_**
Initial stance	96.00	93.69	78.87	96.61	90.81	88.32	90.35	84.42	88.56
Midstance	90.40	90.38	92.66	99.14	99.05	98.60	99.03	95.58	98.95
Terminal stance	95.18	90.59	76.32	95.27	92.85	84.25	88.22	90.47	80.91
Initial swing	72.69	70.30	74.98	98.14	94.62	77.18	92.24	87.67	78.58
Terminal swing	88.73	80.89	80.82	98.63	96.02	91.45	98.41	97.41	81.70
Accuracy	**88.60**	85.17	**80.73**	**97.56**	94.67	**87.96**	**93.65**	91.11	**85.54**
Total Avg	84.83			93.40			90.10		

The bold numbers indicate the minimum and maximum accuracy values of gait recognition in the three load styles and at the three speeds.

**Table 3:** Intra- and inter-load styles and the overall accuracy of gait recognition (%) (mean ± SD) across the three speeds.

	V3	V5	V7	Total
Intra-load style accuracy (mean ± sd)	84.83 ± 3.95	93.40 ± 4.93	90.10 ± 4.15	89.44 ± 5.32
Inter-load style accuracy (mean ± sd)	62.24 ± 6.47	69.05 ± 9.94	72.18 ± 4.67	67.83 ± 8.13
Overall load style accuracy (mean ± sd)	69.77 ± 12.56	77.17 ± 14.70	78.15 ± 9.91	75.03 ± 12.64

## Data Availability

The data presented in this study are available on request from the corresponding author. The data are not publicly available due to restrictions e.g., privacy and ethical.
